# Concise review: Current understanding of extracellular vesicles to treat neuropathic pain

**DOI:** 10.3389/fnagi.2023.1131536

**Published:** 2023-03-03

**Authors:** Kexin Zhang, Pei Li, Yuanyuan Jia, Ming Liu, Jingjing Jiang

**Affiliations:** Department of Anesthesiology, Shengjing Hospital of China Medical University, Shenyang, Liaoning, China

**Keywords:** exosome, neuropathic pain, glial cell, neuroinflammation, noncoding RNA, extracellular vesicles

## Abstract

Extracellular vesicles (EVs) including exosomes are vesicular vesicles with phospholipid bilayer implicated in many cellular interactions and have the ability to transfer multiple types of cargo to cells. It has been found that EVs can package various molecules including proteins and nucleic acids (DNA, mRNA, and noncoding RNA). The discovery of EVs as carriers of proteins and various forms of RNA, such as microRNAs (miRNA) and long noncoding RNAs (lncRNA), has raised great interest in the field of drug delivery. Despite the underlying mechanisms of neuropathic pain being unclear, it has been shown that uncontrolled glial cell activation and the neuroinflammation response to noxious stimulation are important in the emergence and maintenance of neuropathic pain. Many studies have demonstrated a role for noncoding RNAs in the pathogenesis of neuropathic pain and EVs may offer possibilities as carriers of noncoding RNAs for potential in neuropathic pain treatment. In this article, the origins and clinical application of EVs and the mechanism of neuropathic pain development are briefly introduced. Furthermore, we demonstrate the therapeutic roles of EVs in neuropathic pain and that this involve vesicular regulation of glial cell activation and neuroinflammation.

## 1. Introduction

Neuropathic pain is defined as “pain initiated or caused by a primary lesion or dysfunction of the nervous system” by the International Association for the Study of Pain (IASP). Neuropathic pain can be classified into peripheral and central pain based on the sources of the lesion or disease (Scholz et al., 2019). Nociceptors that are defined as primary afferent sensory neurons can be activated by noxious stimuli and transmit pain signals to SDH (Basbaum et al., 2009). The imbalance between synaptic excitation and inhibition in the SDH leads to aberrant somatosensory signals which are subsequently conveyed to the brainstem and higher brain regions (Tsuda et al., 2017). Up to 8% of the general population suffer from neuropathic pain, which is refractory to recent clinical therapies and seriously affects their quality of life. Therefore, novel, efficacious treatment strategies are urgently needed, but the exact molecular and cellular mechanisms underlying neuropathic pain remain unclear. A large part of cell–cell communications and molecular signaling pathways come into play during regulation of the sensitization of nociceptive pathways. The development and maintenance of neuropathic pain is coupled with the interactions between neurons-glial cells, and neuronal-immune cells (Vicario et al., 2020; Yu et al., 2020b).

EVs were identified as particles naturally released from the cell that are delimited by a lipid bilayer and cannot replicate (Théry et al., 2018). EVs are classified typically based on their size, biogenesis, and biophysical properties: exosomes, microvesicles and apoptotic bodies, many of which show pleiotropic biological functions (Rufino-Ramos et al., 2017; O’Brien et al., 2020). Exosomes are a specific EVs subtype which are considered to be of endosome-origin and ∼30 to ∼200 nm in diameter (Pegtel and Gould, 2019). EVs were initially thought to function only as cellular ‘garbage’ disposals, but with accumulating evidence concerning their origin, composition and transportation, and intercellular signal transmission, it has become clear that EVs have multiple functions. EVs that mediate autocrine, paracrine, and endocrine effects are taken up by surrounding cells or circulate in the blood and are eventually taken up by distal cells (El Andaloussi et al., 2013). EVs with different cargo act as master switches orchestrating both immune and neuronal processes, suggesting that they are involved in many pathophysiological processes such as neuropathic pain (Barile and Vassalli, 2017; Sosanya et al., 2020). EVs derived from different cells contain different cargo compositions including nucleic acids, proteins, lipids, amino acids, and metabolites and participate in numerous physical and pathological processes (Zhang Y. et al., 2019). Studies found that exosomes secreted from specific areas are involved in human neuropathic pain conditions such as mPFC and NAc can enhance allodynia in mouse CCI models (Yu et al., 2020a). In this review, we explore the molecular and cellular mechanisms and therapeutic potential of EVs for neuropathic pain. Because of the hard differentiation, the exosome terminology is used if clarified in the references and the term EV is used if the differentiation is unclear here.

## 2. Extracellular vesicles

### 2.1. Biogenesis of EVs

**Figure 1:**
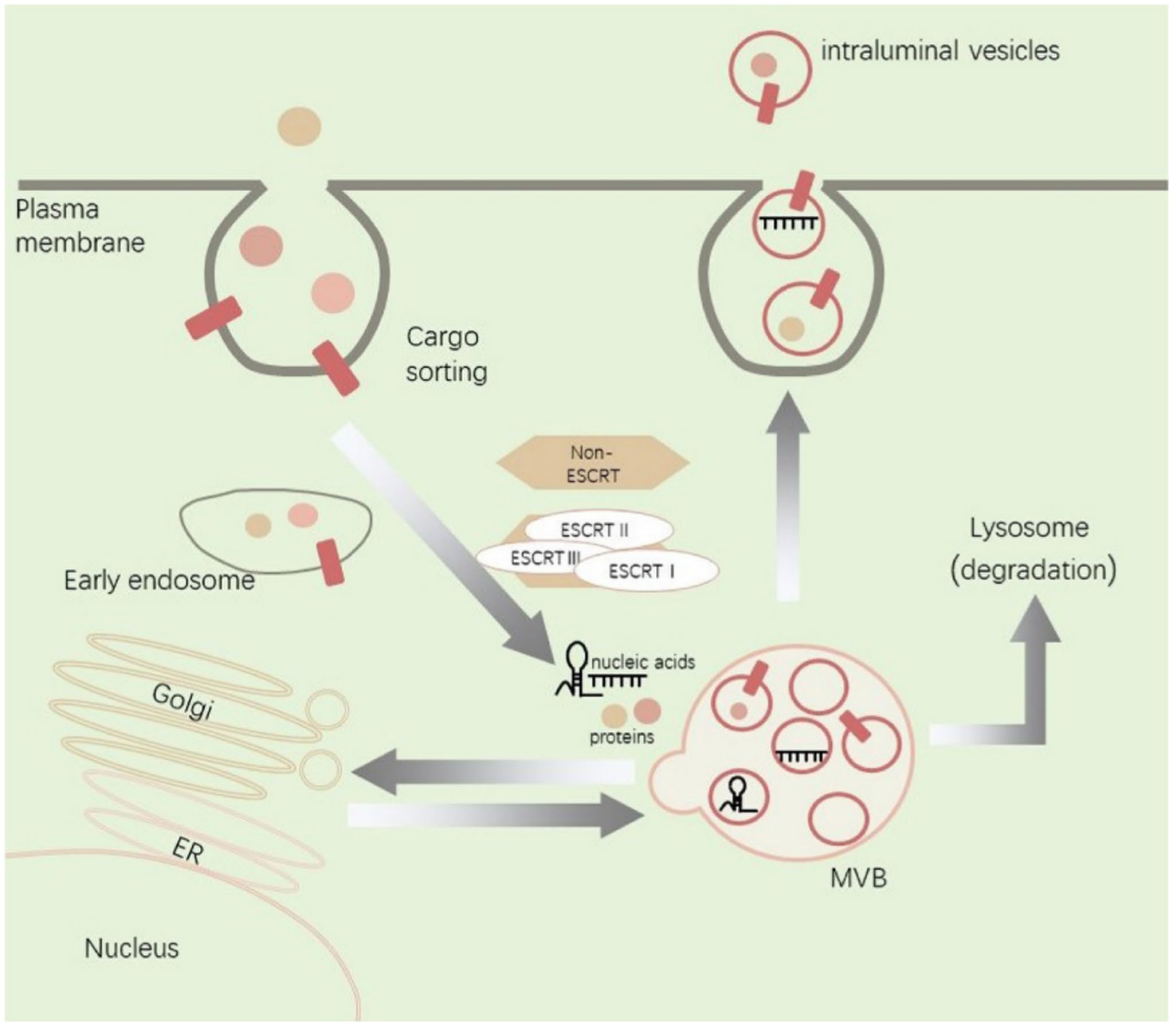
Illustration of EVs biogenesis, cargo sorting, and release.

EVs are formed when the plasma membrane double invaginates and eventually generates the MVB, which can be degraded in lysosomes or fuse with cell membranes to release inclusions as EVs (Bebelman et al., 2018; Kalluri and LeBleu, 2020). The biogenesis and transport of EVs are associated with a large number of proteins, including ESCRT, Ras-related proteins GTPase Rab, ceramide, and phospholipids (Trajkovic et al., 2008; Mathieu et al., 2019). Exosomes are made of lipid bilayer structures and appear biconcave or cup-shaped when produced by artificially drying during TEM (Yellon and Davidson, 2014; Lobb et al., 2015). Bilayer lipid vesicles in solution are nearly spherical. The method of purification and processing for EM can lead artifactual observation of different shapes (Chernyshev et al., 2015). EVs generated by cells including neurons, glial cells and immune cells can promote cell-to-cell communication and regulate biological processes after nerve injury (López-Leal et al., 2020). In addition, MSCs including bone marrow MSCs, gingival MSCs, adMSCs, and hucMSCs can release EVs to regulate the pathological progression after nerve injury (Bryk et al., 2022; Zhang Y. U. et al., 2022). EVs derived from different cells contain different signaling molecules and surface antigens as well as different cargos including unique proteins, lipids, and genetic material (Toh et al., 2018). This intercellular communication involves two different mechanisms an ESCRT-dependent mechanisms and an ESCRT-independent mechanisms (D'Agnelli et al., 2020). EVs can affect phenotypic and molecular alterations of receptor cells by direct or indirect delivery of signaling molecules to receptor cells through binding with cell surface receptors and receptor-mediated endocytosis or direct membrane fusion (Meldolesi, 2018; Van Niel et al., 2018; Figure 1).

Within the endosomal system, cargos including proteins and nucleic acids are internalized from extracellular space. Cargos are sorted into early endosomes and then mature into MVB through a process that involves interactions with the Golgi complex. MVB can be degraded in lysosomes or fuse with cell membranes to release inclusions as EVs. The biogenesis and transport of EVs are associated with ESCRT and non-ESCRT. ER, endoplasmic reticulum; MVB, multivesicular bodies.

### 2.2. Clinical application of EVs

As biomarkers for diagnose and treatments, EVs are at an emerging stage of clinical application. EVs mediating intercellular communication trigger immune responses, inflammatory responses, and many other processes (Harrell et al., 2019; Qiu et al., 2021; Ashrafizadeh et al., 2022). Exosomes presenting in biological fluids and carrying specific membrane proteins are internalized by recipient cell through soluble and juxtacrine signaling events, fusion, and receptor/raft-mediated endocytosis, as well as phagocytosis, and regulate multiple physiological and pathological processes (Hu et al., 2020). The characteristic properties of exosomes during delivery of functional cargos is that they can target specific cells, which also favors their use as therapeutic vehicles (Hessvik and Llorente, 2018). Exosomes affect neuroinflammation and neuropathic pain by delivering proteins, mature miRNAs, and translatable transcripts (Ramanathan and Ajit, 2016). MiR-25-3p, miR-320B, miR-93, miR-126-3p, and RNU48 from serum-derived exosomes in patients with complex regional pain syndrome show significant differential expression (McDonald et al., 2014). Carrying miR-181c-5p containing exosomes repress CCI-induced neuropathic pain through inhibition of neuroinflammation (Zhang Y. U. et al., 2022), which provided the therapeutic rationale for studying EVs to treat neuropathic pain.

## 3. Pathogenesis of neuropathic pain

Neuropathic pain constitutes a significant proportion of all chronic pain suffers. Most patients with neuropathic pain complain of spontaneous pain, hyperalgesia (abnormal hypersensitivity to stimuli), and allodynia (nociceptive responses to non-noxious stimuli under normal states). Studies have shown that the disappearance of myelin breakdown after peripheral nerve injury-induced inflammation is critical for axon regeneration (Ying et al., 2018). Their presence impedes nerve repair. The pain signals are transmitted into the central nervous system, including the SDH by the primary afferent nociceptors on DRGs (Navarro et al., 2007; Chen et al., 2020). Moreover, with SDH synthesizing and releasing a variety of classical inflammatory mediators, nociceptor sensitization leads to ectopic firing and erroneous conduction velocities which changes the sensitization of supraspinal nociceptor neurons through ascending transmission, and finally causes neuropathic pain (Macone and Otis, 2018). The sprouting nociceptive fibers terminate in an unusual way in the denervated skin, with significant allodynia occurring months after nerve injury (Gangadharan et al., 2022). Evidence has added to a growing appreciation of the complex link between neuropathic pain and nerve regeneration (Xie et al., 2017). Changes in ion channels, activation of immune cells, compounds released by glial cells, and epigenetic modifications are all involved in neuropathic pain (Finnerup et al., 2021). Furthermore, mast cells, macrophages, neutrophils, and their released mediators are associated with neuropathic pain. Agtr2-expressing macrophages at the site of nerve injury triggers neuropathic pain caused predominantly by immune cells (Shepherd et al., 2018).

Neuroinflammation which is a localized form of inflammation in the peripheral nervous and central nervous systems is becoming increasingly implicated in neuropathic pain (Ji et al., 2014; Jiang et al., 2020). In the DRG, nerve injury not only causes neuronal changes but also leads to the activation of peripheral glial cells such as Schwann cells and satellite glial cells, eventually leading to neuroinflammation and neuropathic pain (Ji et al., 2013; Yuan et al., 2020). Activated central glial cells also cause diverse alterations that change neural excitability, leading to the development of neuropathic pain (Kohno and Tsuda, 2021).

The discovery of extensive epigenetic regulation such as noncoding RNA modification, DNA methylation, and histone acetylation provides a novel perspective on the mechanism of neuropathic pain. Noncoding RNAs, are an important class of RNA molecule that typically does not encode detectable proteins, may regulate neuropathic pain *via* multiple and complex mechanisms. Peripheral noxious stimuli drives expressional changes in noncoding RNAs, such as miRNA, lncRNA, and circRNA, and these changes are associated with the aberrant expression of its target mRNA and the occurrence and development of neuropathic pain (Li L. et al., 2019; Zhang S. B. et al., 2019). Specifically, miRNA and lncRNA can be functionally shuttled between cells *via* exosomes and are termed exosomal shuttle RNA (Valadi et al., 2007), and how these exosomes function as exosomal shuttle RNA carriers in neuropathic pain represents our next research question.

## 4. Extracellular vesicles regulate neuropathic pain

### 4.1. Extracellular vesicles regulate neuropathic pain *via* glial cell activation

Glial cells, also known as neuroglia, modulate neurotransmission at the synaptic level. Now it has been found that glial cells play an important role in neuroinflammation and neuropathic pain (Tsuda and Inoue, 2016; Hirbec et al., 2020). Glial cells are located in DRGs, spinal cord and brain, and show a series of changes such as a shifting phenotype and release of inflammatory mediators in response to a damaging signal within the nervous system to drive central and peripheral sensitization and participate in neuropathic pain evolution (Chen et al., 2018; Matsuda et al., 2019).

Microglia (macrophages residing in the central nervous system) are sensors for the detection of abnormal alterations in response to internal and external insults in the central nervous system (Ji et al., 2016). Studies have found that in SCI models exosomes were delivered to the injury site and significantly enhance axonal regeneration, reduce cell apoptosis, and reduce activation of microglia and astrocytes (Guo et al., 2019). MSC-derived EVs can promote phenotype transformation and function of macrophages/microglia from pro-inflammatory M1 to anti-inflammatory M2 types. The activation of glial cells requires the participation of pro-inflammatory factors which may be related to miRNAs in exosomes, such as miR-34a-5p, miR-21, and miR146a-5p (Domenis et al., 2018). Previous studies have shown that TBI is associated with neuropathic pain (Leung, 2020). Exosomes mediate neuron-glial cell intercellular communication and following TBI, microglia reduce the release of exosomes containing miR-5,121 to inhibit neurite outgrowth and synapse recovery of neurons (Zhao et al., 2021).

The expression of lncGm37494 targeting miR-130b-3p to regulate microglia polarization is increased in exosomes secreted by ADSCs under hypoxia, and has been suggested that exosomes can repair SCI by delivering lncGm37494 (Shao et al., 2020). Intravenous administration of hucMSC-derived exosomes significantly inhibits the transcription levels of astrocytes and microglia, suggesting a reduced inflammatory state from SCI (Kang and Guo, 2022), and single and continuous intrathecal infusion of hucMSC-derived exosomes after SNL can prevent and reverse neuropathic pain. Moreover, it has been found that the analgesic effects of exosomes may involve their actions on neurons and glial cells (Shiue et al., 2019).

**Figure 2:**
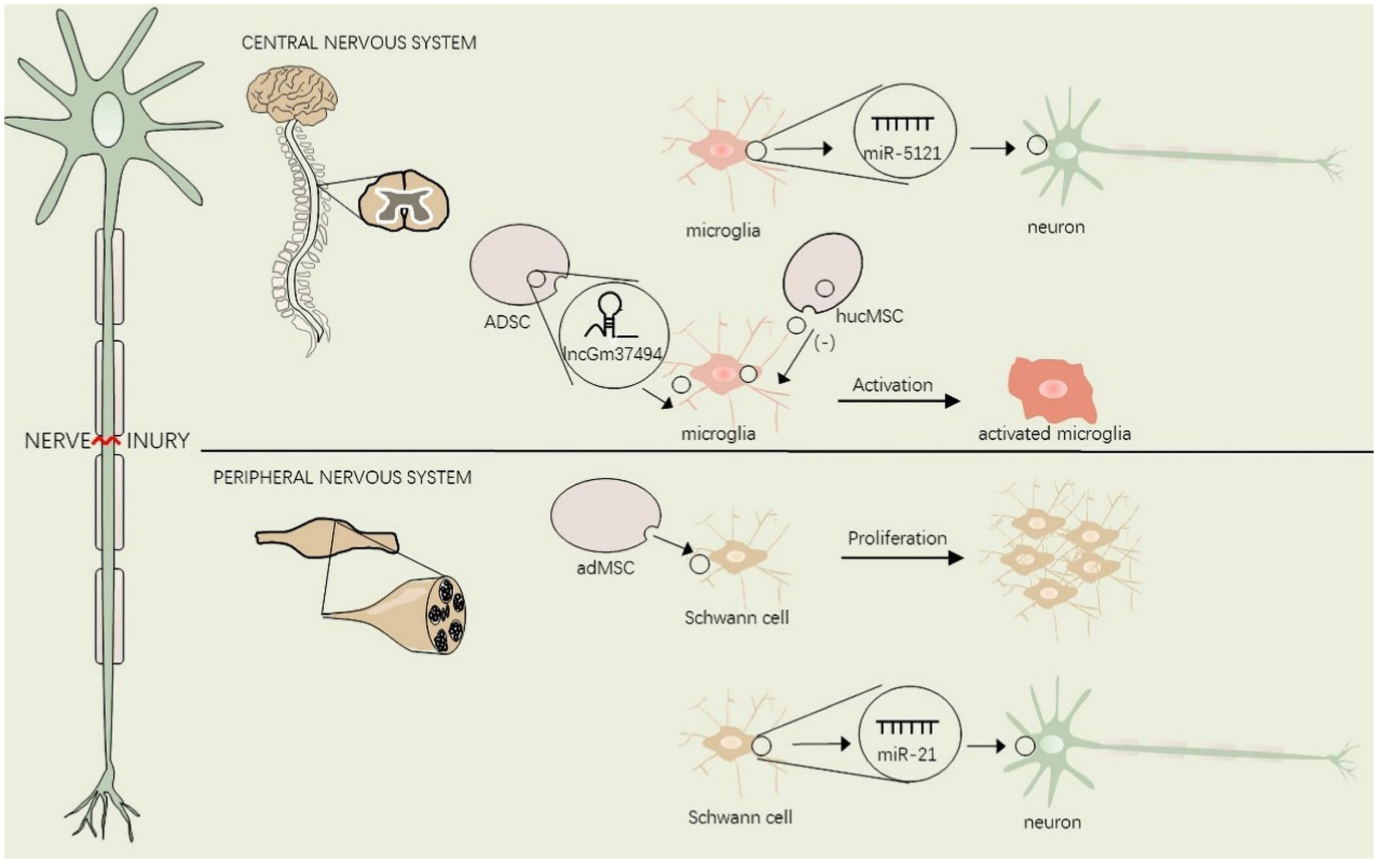
EVs regulate glial cell activation in the central and peripheral nervous system after nerve injury.

As the most abundant glial cell in the peripheral nervous system, Schwann cells undergo phenotypic modulation, proliferate and interact with nociceptive neurons by releasing glial mediators (growth factors and cytokines) after nerve injury. These dramatic alterations in Schwann cells promote nerve regeneration and eventually influence neuropathic pain (Dubový, 2011; Wei et al., 2019). During neuropathic pain, many receptors on Schwann cells are differentially expressed, such as P2X4R, HCAR2 (Boccella et al., 2019; Su et al., 2019). Recent studies have proved the presence of growth factors such as BDNF, NGF, FGF-1, IGF-1, and GDNF in adMSC exosomes. Furthermore, adMSCs-derived exosomes which internalized into the Schwann cells exhibited the ability to promote Schwann cell proliferation *in vitro* and enhance nerve regeneration following SNI *in vivo* (Bucan et al., 2019), and interestingly, BDNF and NGF have been identified to correlate with neuropathic pain (Dai et al., 2020; Wu et al., 2021). In addition, exosomal miR-21 was shown to promote the Schwann cell proliferation and the expression of NGF, BDNF, and GDNF after peripheral nerve injury. And exosomal miR-21 secreted by Schwann cells can promote neurite outgrowth and functional repair after SNI (Liu Y. P. et al., 2022; Figure 2).

Lesions of the nervous system from the peripheral nociceptor to the spinal cord and brain may give rise to neuropathic pain. This figure shows the interaction between EVs and glial cells in the central and peripheral nervous system. adMSC, adipose-derived mesenchymal stem cells, hucMSC, human umbilical cord mesenchymal stem cells, ADSC, Adipose tissue-derived mesenchymal stem/stromal cells.

### 4.2. Extracellular vesicles regulate neuropathic pain *via* neuroinflammation

Neuroinflammatory activation of glial cells and immune cells after nerve injury is being increasingly implicated in neuropathic pain (Chambel et al., 2020; Karri et al., 2022). EVs pose tremendous anti-inflammatory capacity following nerve injury and the roles of EVs in neuropathic pain will be a particularly interesting area of future inquiry.

EF-MSCs secrete EVs to repress NLRP3 inflammasome activation and to inhibit the expression of inflammatory cytokines related to neuroinflammation after SCI (Huang et al., 2020). Macrophages change their polarization to anti-inflammatory phenotype which can effectively control the development of inflammation (Ma et al., 2020). HucMSC-derived exosomes can stimulate bone marrow-derived macrophages to transform into M2 type, which can also inhibit inflammation through decreasing inflammatory factors such as TNF-α, MIP-1α, IL-6 and IFN-γ (Sun et al., 2018). MSC derived exosomes can relieve microglia/macrophage and astrocyte-mediated neuroinflammation in rats after TBI (Zhang et al., 2020). Studies suggest a critical role for proteins in the mediation of signaling mechanisms underlying neuropathic pain after SNI. Furthermore, the compliment protein C5a and ICAM-1 were found to be significantly up-regulated in EVs purified from SNI mouse serum (Jean-Toussaint et al., 2020). C5a can help induce the expression of inflammatory factors in microglia and promote the development of their inflammatory status (Liu et al., 2018). Furthermore, C5a and its receptor C5aR can simultaneously participate in the process of neuropathic pain (Quadros and Cunha, 2016).

**Figure 3:**
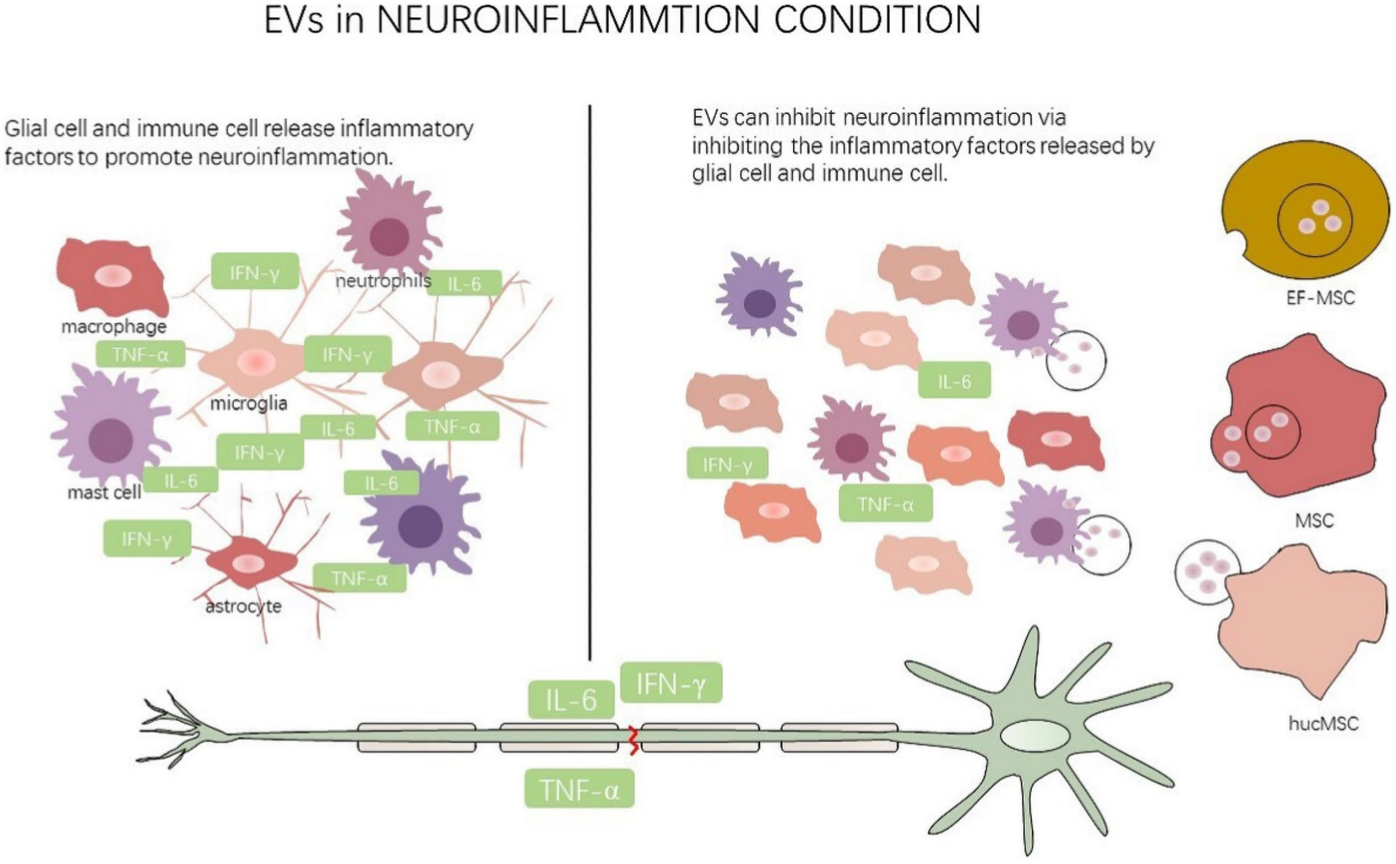
EVs regulate neuroinflammation state.

MSC-derived EVs can facilitate macrophage/microglia polarization from M1 to M2 phenotype. miR-21 and miR-19b in EVs derived from differentiated PC12 cells and MSCs can suppress apoptosis of neurons by decreasing the expression of PTEN which can be applied during post-SCI repair (Xu et al., 2019). We found that miR-216a-5p inside MSC-derived exosomes targeted TLR4 and shifted this M1/M2 polarization to inhibit neuroinflammation after SCI which was mediated by microglial activation (Liu et al., 2020). Exosomes derived from huc-MSC were found to deliver miR-199a-3p/miR-145-5p to neurons causing attenuation of the release of inflammatory factors and promotion of neuronal neurite outgrowth following SCI (Wang et al., 2021). circRNA is a type of noncoding RNA that forms a covalently closed continuous loop. Many of them are enriched in EVs, but whether EV circRNAs regulate neuropathic pain is still a shortage of investigation (Marangon et al., 2023). Circ_003564, a recently discovered circRNA, acted as a critical effector in BMSC-exosome-mediated pyroptosis in the SCI model and these exosomes containing circ_003564 may contribute to treatment efficacy in SCI (Zhao et al., 2022). lncRNA H19 in BMSC derived exosomes can further regulate microglia polarization to release neuroinflammatory signals *via* sponging miR-29b-3p (Zong et al., 2021). Significantly, the treatment using lncRNA TCTN2-modified exosomes significantly improved functional *in vivo* recovery in SCI rats and alleviated LPS-induced inflammation *in vitro* (Liu J. et al., 2022; Figure 3).

This figure illustrates that multiple cells secrete EVs to glial cell and immune cell to mediate neuroinflammation after nerve injury. EF-MSC: epidural fat-mesenchymal stem cells, MSC: mesenchymal stem cells, hucMSC: human umbilical cord mesenchymal stem cells.

### 4.3. Extracellular vesicles regulates neuropathic pain *via* miRNA

Diverse evidence has shown that RNA molecules inside EVs are generally less than 300 nt in length which is smaller than human mRNA, indicating a role for noncoding RNA (Chen et al., 2010). Furthermore, miRNAs represent an important cargo type in EVs can obstruct the expression of target genes by inhibiting target mRNA translation and accelerate mRNA degradation. These miRNA are protected from RNase by the exosomes or cellular membranes even in extreme cases (Koga et al., 2011). The miRNAs enriched in EVs are phagocytosed by receptor cells and can mediate cell-to-cell communication thus participating in the occurrence and maintenance of neuropathic pain (Sosanya et al., 2020).

The expression levels of miR-21 in exosomes derived from blood serum is significantly increased in the pSNL model, and exosomal miR-21 is likely to be a diagnostic biomarker for neuropathic pain (Hori et al., 2016). This confirmed that SNI induced upregulation of miR-21-5p, let7b, miR-124, and miR-134 expression in the lumbar DRG and DRG sensory neurons can release exosomes containing miR-21-5p which were subsequently internalized by macrophages and converting them to the M1 type. The study also indicated that intrathecal injection of the miR-21 antagomir inhibits hyperalgesia and relieves neuropathic pain (Simeoli et al., 2017).

Intravenous and intrathecal administration of MSCs effectively relieves neuropathic pain following CCI or SCI (Yousefifard et al., 2016; Al-Massri et al., 2019) and MSC exosome therapy has shown the same functions as MSCs therapy, such as anti-inflammation, regulation of the immune system, and regulation of cell differentiation (Lee et al., 2021). In CCI rats, miR-181c-5p from MSC-derived exosomes were found to be significantly downregulated in a time-dependent manner. Intrathecal administration of exosomal miR-181c-5p which targets microglia and inhibits the secretion of inflammatory factors alleviated neuropathic pain and the neuroinflammatory response after CCI (Zhang Y. U. et al., 2022).

In the CCI model, downregulated miR-183 in the sciatic nerve targeting transcription factor FoxO1 and tight junction protein claudin-5 promoted mechanical hypersensitivity. Exosomal miR-183 was found decreased from sera of complex regional pain syndrome patients and can regulate neuropathic pain state *via* regulating microvascular barrier (Reinhold et al., 2022). In conclusion, our study assesses exosomal noncoding RNAs as one modifier of neuropathic pain (Table 1).

**Table 1:** Exosomal noncoding RNAs regulate neuropathic pain.

Specific conditions	miRNA in exosomes	Site	Noncoding RNAs’ contributions	Regulatory mechanism	Reference
SNI	miR-21-5p	DRG	Exosomal miR-21-5p was internalized by macrophages and polarized macrophages to M1 type.	Noncoding RNAs regulate glial cell activation.	Simeoli et al. (2017)
SCI	lncGm37494	ADSC	lncGm37494 in exosomes targeted miR-130b-3p to regulate microglia polarization.		Shao et al. (2020)
SCI	miR-216a-5p	MSC	Exosomal miR-216a-5p targeted TLR4 and shifted M1/M2 polarization to inhibit neuroinflammation.	Noncoding RNAs regulate neuroinflammation.	Liu et al. (2020)
SCI	miR-199a-3p/ miR-145-5p	huc-MSC	Exosomal miR-199a-3p/ miR-145-5p were internalized by neurons to attenuate the release of inflammatory factors.		Wang et al. (2021)
SCI	circ_003564	BMSC	Exosomal circ_003564 was internalized by neurons to relieve pyroptosis.		Zhao et al. (2022)
LPS	lncRNA H19	BMSC	Exosomal lncRNA H19 regulates microglia polarization to relieve neuroinflammation *via* sponging miR-29b-3p.		Zong et al. (2021)
SCI	lncRNA TCTN2	MSC	lncRNA TCTN2 improved functional recovery of SCI rats by miR-329-3p/IGF1R axis.		Liu J. et al. (2022)
CCI	miR-183	Sera	Decreased sera exosomal miR-183 contributed to neuropathic pain state *via* regulating microvascular barrier.	Noncoding RNAs regulate neuropathic pain	Reinhold et al. (2022)
CCI	miR-181c-5p	MSC	Exosomal miR-181c-5p targeted microglia and inhibited the secretion of inflammatory factors to alleviate neuropathic pain.		Zhang Y. U. et al. (2022)
SNI	miR-21	Schwann cell	Exosomal miR-21 was upregulated to promote Schwann cell proliferation after peripheral nerve injury.		Liu Y. P. et al. (2022)

## 5. Conclusion and perspectives

The main manifestations of neuropathic pain such as spontaneous pain, hyperalgesia, and hyperalgesia, will last for long periods (Kuner and Flor, 2016). Primary sensory neurons show alternations in ion channels and pain-related gene expression, and after receiving noxious stimuli in the peripheral nervous system. Then pain signals are transmitted upwards to the spinal cord and brain, resulting in central sensitization which is an increased responsiveness to nociceptive neurons to normal or subthreshold afferent input in the central nervous system (Zhang J. et al., 2021). Currently there is no general consensus for the underlying mechanism of neuropathic pain as it is complex and multifactorial. In recent years cell transplantation therapy has received widespread attention in the clinical literature. Pluripotent stem cell-derived GABAergic neuron transplants were shown to relieve neuropathic pain induced by both peripheral and central nerve injuries (Dugan et al., 2020; Manion et al., 2020). However, concerns have been raised about the safety of cell transplantation therapy for clinical use, because cell-therapy presents inherent risks, such as microvascular obstruction, malignant tumor formation, and high cost (Jeong et al., 2011; Lou et al., 2017). The occurrence and development of neuropathic pain is accompanied by intercellular signal transduction. EVs were demonstrated to promote cell activation, regulate neuroinflammation and immune response, and promote angiogenesis and axonal growth after nerve injury (Yu et al., 2021). Exosomes, representing a subtype of EVs, have also been a popular studied paracrine topic during the past two decades and are used as a treatment strategies for neuropathic pain (Dong et al., 2019). Exosomes are efficient and safe nanocarriers for pain-targeted gene therapy *via* intranasal delivery and tail vein injection because of their small molecular weight, ease of transit across the blood–brain barrier and other advantages (Sun et al., 2018; Guo et al., 2019). The use of exosomes could radically eliminate the risk of rejection and offer substantial neuroprotective effects with potential immunoprotective and anti-inflammatory roles (Shojaei et al., 2019).

EVs hold great potential for clinical application with their excellent biocompatibility and bi-layered lipid structures, but the clinical application of EVs faces many challenges, including low yield, impurity and loading efficiency (Tran et al., 2020). Engineered EVs carrying therapeutic molecules hold promise as alternative therapies. The barriers to translation of exosomes remain the difficulty to precisely target the specific cell while limiting off-target biodistribution and the presence of naturally incorporated cellular genetic impurities with potential immunogenicity (Ha et al., 2016; Bunggulawa et al., 2018; Gurung et al., 2021). Specific, loosely associated, proteins and lipids may be lost or masked during exosome isolation, and typical isolation protocols are not specific for exosomes which may influence their targeting behaviors (Kooijmans et al., 2016). With the expanded interest in the field, studies focus on an easy and efficient strategy to broaden, alter or enhance exosome targeting capability. Previous reports have suggested that surface modifications can increase the delivery ability of exosomes (Armstrong et al., 2017; Tian et al., 2018). The low yield of exosomes is a bottleneck for clinical translation. It has shown that knocking down of Rab4 gene and optimization of culture conditions such as supplementing red cell membrane particles can augment the production of exosomes per cell without sacrificing the therapeutic efficacy (Zhang R. et al., 2022).

Glial cells have been found to be a vital component of neuropathic pain and are widely activated during the pain conditions. It is demonstrated that glial cells proliferate, change their morphology and function, change the expression of pain-related genes, and release diffusible factors to affect other cells after a noxious stimulation (Li T. et al., 2019). Activated glial cells release inflammatory factors, such as TNF-α and IL-6, to promote neuroinflammation, and lead to constant painful irritation of the sensory nerves. Studies have provided evidence that microglia can shed new light on the underlying molecular and cellular mechanisms of neuropathic pain (Inoue and Tsuda, 2018). EVs secreted by peripheral macrophages regulate microglial polarization and increase microglia autophagy after SCI *via* the PI3K/AKT/mTOR signaling pathway (Zhang B. et al., 2021).

As a hot topic in epigenetic research, noncoding RNAs can be encapsulated by EVs and target receptor cells in a paracrine manner to regulate a variety of diseases processes. Recently, several studies have demonstrated that vesicular miRNAs can regulate neuropathic pain. Exosome-encapsulated miR-21 can regulate the occurrence and development of neuropathic pain (Simeoli et al., 2017). Compelling evidence supports the existence of lncRNA and circRNA in body fluids have further clinical implications (Wang et al., 2019; Liu J. et al., 2022). However, the effects and clinical application of vesicular lncRNA and circRNA in neuropathic pain need further exploration.

## Author contributions

KZ conceived the idea and wrote the initial draft of the manuscript. PL and YJ prepared the figures and table. ML and JJ performed the analysis with constructive discussions. All authors read and approved the final manuscript.

## Funding

The work was supported by Natural Science Foundation of Liaoning Province (2022-YGJC-37) and 345 Talent Project of Shengjing Hospital.

## Conflict of interest

The authors declare that the research was conducted in the absence of any commercial or financial relationships that could be construed as a potential conflict of interest.

## Publisher’s note

All claims expressed in this article are solely those of the authors and do not necessarily represent those of their affiliated organizations, or those of the publisher, the editors and the reviewers. Any product that may be evaluated in this article, or claim that may be made by its manufacturer, is not guaranteed or endorsed by the publisher.

## Abbreviations

EVs: extracellular vesicles
ESE: early-sorting endosome
MVBs: multivesicular bodies
CCI: chronic constriction injury
SCI: spinal cord injury
SNI: spared nerve injury
ICAM-1: intercellular adhesion molecule-1
adMSCs: adipose-derived mesenchymal stem cells
ADSC: Adipose tissue-derived mesenchymal stem/stromal cells
EF-MSC: epidural fat-mesenchymal stem cells
NF200: neurofilament protein 200
GAP-43: growth-associated protein-43
hucMSC: human umbilical cord mesenchymal stem cells
mPFC: medial prefrontal cortex
NAc: nucleus accumbens
TNF-α: tumor necrosis factor-alpha
IL-6: interleukin-6
SDH: spinal dorsal horn
TBI: traumatic brain injury
AT2R: type 2 angiotensin II receptor
NGF: Nerve growth factor
BMSC: bone marrow mesenchymal stem cell
TEM: transmission electron microscopy
circRNA: circular RNA
